# Designing and Evaluating Interventions to Halt the Transmission of Tuberculosis

**DOI:** 10.1093/infdis/jix320

**Published:** 2017-11-03

**Authors:** David W Dowdy, Alison D Grant, Keertan Dheda, Edward Nardell, Katherine Fielding, David A J Moore

**Affiliations:** 1 Department of Epidemiology, Johns Hopkins Bloomberg School of Public Health, Baltimore, Maryland;; 2 Division of Global Health Equity, Harvard Medical School, Brigham and Women’s Hospital, Boston, Massachusetts;; 3 TB Centre and; 4 Department of Infectious Disease Epidemiology, London School of Hygiene and Tropical Medicine, United Kingdom; and; 5 Africa Health Research Institute, School of Nursing and Public Health, University of KwaZulu-Natal, Durban,; 6 School of Public Health, Faculty of Health Sciences, University of the Witwatersrand, Johannesburg, and; 7 Division of Pulmonology, Department of Medicine, University of Cape Town, South Africa

**Keywords:** Tuberculosis, disease transmission, infectious, public health, prevention and control, diagnosis, infection control

## Abstract

To reduce the incidence of tuberculosis, it is insufficient to simply understand the dynamics of tuberculosis transmission. Rather, we must design and rigorously evaluate interventions to halt transmission, prioritizing those interventions most likely to achieve population-level impact. Synergy in reducing tuberculosis transmission may be attainable by combining interventions that shrink the reservoir of latent *Mycobacterium tuberculosis* infection (preventive therapy), shorten the time between disease onset and treatment initiation (case finding and diagnosis), and prevent transmission in key settings, such as the built environment (infection control). In evaluating efficacy and estimating population-level impact, cluster-randomized trials and mechanistic models play particularly prominent roles. Historical and contemporary evidence suggests that effective public health interventions can halt tuberculosis transmission, but an evidence-based approach based on knowledge of local epidemiology is necessary for success. We provide a roadmap for designing, evaluating, and modeling interventions to interrupt the process of transmission that fuels a diverse array of tuberculosis epidemics worldwide.

Over the past 15 years, the number of deaths due to tuberculosis has fallen by 22%, but the annual number of incident cases of tuberculosis has remained the same (Figure 1) [1]. During this time, many countries have witnessed sustained declines in tuberculosis incidence, but in most high-burden countries, the estimated annual risk of *Mycobacterium tuberculosis* infection remains largely unabated [2]. To make substantial progress in reducing the global incidence of tuberculosis, we must better understand the dynamics of tuberculosis transmission and develop a comprehensive strategy for halting it. Such a strategy could include innovative interventions designed to interrupt tuberculosis transmission, rigorous evaluation of those interventions, and mathematical models to prioritize interventions with the greatest potential to achieve population-level impact.

**Figure 1.**
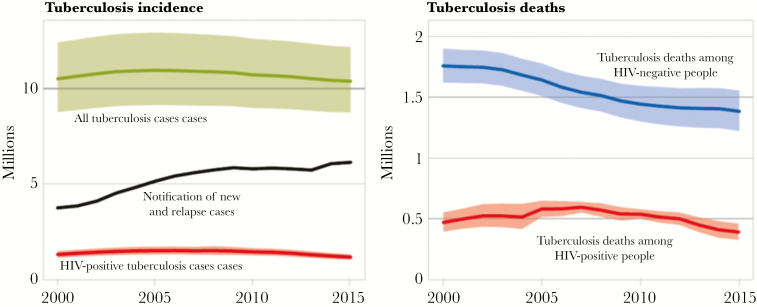
Estimated global tuberculosis incidence and mortality. Whereas mortality due to tuberculosis has been falling steadily over the past 15 years, incidence has remained relatively constant, pointing to the likelihood that interventions to reduce tuberculosis-associated mortality (through better passive diagnosis and treatment) have had less impact on transmission. Shaded areas represent uncertainty intervals. Abbreviation: HIV, human immunodeficiency virus. Reproduced from [1] with permission of the World Health Organization.

Tuberculosis transmission can be halted. In Bethel, Alaska, a combination of case finding, treatment, and preventive therapy reduced the annual risk of *M. tuberculosis* infection in children from 24.6% to 1.1% in 10 years [3]. In Providence and Letitia Hill, Peru, a similar set of interventions reduced tuberculosis incidence by >75% and new infections among children by 62% over 2 years [4]. In both cases, the effects of these transmission-halting interventions were sustained for decades into the future. The long latency period associated with *M. tuberculosis* infection [5] provides a uniquely long window of opportunity to interrupt tuberculosis transmission by preventing reactivation. Interventions to interrupt transmission also include identifying and treating individuals at early disease stages [6] and engineering the built environment to reduce transmission in high-risk settings.

In considering possible transmission-halting interventions, the principles of R_0_, the basic reproduction number, are useful (Figure 2). R_0_ is the product of 3 components: the contact rate (*c*), the probability of transmission per contact (*β*), and the duration of infectiousness (*d*). Interventions to prevent tuberculosis progression diminish *c* (and *d*) by reducing the community-wide burden of infectiousness. Interventions to find and treat active cases at earlier stages of disease diminish *d*. Interventions on the built environment aim to diminish *β*. Since these 3 components multiply to produce R_0_, combining these corresponding 3 types of interventions can have a synergistic effect in halting tuberculosis transmission. Here we provide a roadmap for designing, evaluating, and modeling interventions to halt tuberculosis transmission and thus interrupt the underlying process fueling the diverse array of tuberculosis epidemics worldwide.

**Figure 2.**
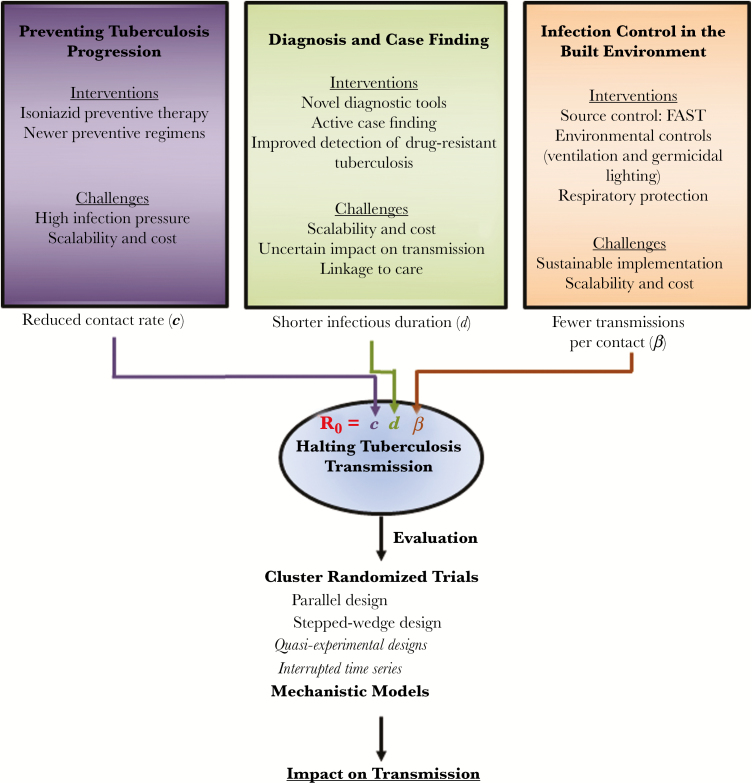
A roadmap to halting tuberculosis transmission. Interventions to prevent progression, improve diagnosis and case finding, and reduce infection in settings such as the built environment operate synergistically to reduce the basic reproductive number (R_0_) of tuberculosis and help halt tuberculosis transmission. Rigorous evaluation of these interventions is critical to ensure impact.

## POPULATION-LEVEL INTERVENTIONS TO PREVENT TUBERCULOSIS PROGRESSION

Isoniazid preventive therapy (IPT) reduces the risk of tuberculosis by 60% at the individual level [7] and has also been studied as part of a package to halt transmission at the population level. Cluster-randomized trials in the late 1950s in Alaska [8], Greenland [9], and Tunisia [10] investigated the effect of population-wide IPT in settings where active disease was so common that everyone could be considered a contact [11]. The aim of these trials was not explicitly to interrupt transmission but to reduce the reservoir of latent *M. tuberculosis* infection [11]. To avoid giving preventive therapy to those with tuberculosis, each trial was preceded by population-based radiological screening, with coverage of “most” individuals school aged and older in Alaska, 90% of adults in Greenland, and 95% of the population in Tunisia [8, 9, 11]. Pretreatment loss to follow-up among individuals with tuberculosis was not quantified but was likely low; in Alaska and Greenland, treatment delivery in sanatoria likely resulted in high completion.

In Alaska, households were randomized to IPT or placebo groups for 1 year, with all household members receiving the same study drug, to allay concerns about pill sharing [8]. Cumulative tuberculosis incidence was lower in intervention households, compared with control households, over a 6-year period (1.90% vs 4.67%). The highest tuberculosis incidence rates and greatest reduction with IPT were among individuals with “inactive” (and not previously treated) *M. tuberculosis* infection detected on chest radiography [8], suggesting that an important mechanism of the intervention may have been to reduce reactivation of “inactive” *M. tuberculosis* infection. In Greenland, trial clusters were villages (to simplify delivery) [9] and in Tunisia were city blocks [10]. In contrast to Alaska, these 2 trials reported no benefit of IPT, a finding that was attributed to an inadequate isoniazid dose or poor treatment adherence (although a reanalysis of Greenland trial data [12] suggested that use of IPT was associated with a one-third reduction in incidence during the first 6 years of follow-up).

**Figure 3.**
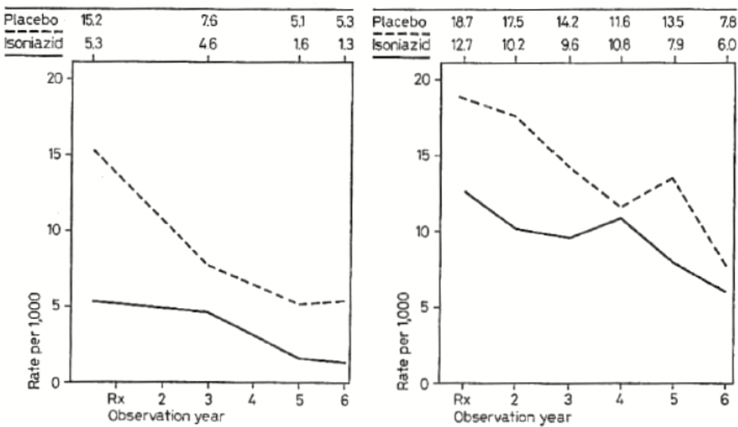
Reductions in tuberculosis incidence after trials of preventive therapy. In both Alaska (left) and Greenland (right), the tuberculosis incidence fell dramatically in both the isoniazid and placebo arms following the implementation of active case finding, linkage to effective treatment, and (in the isoniazid arms) preventive therapy. Reproduced from [11]. Permission to reprint this figure has been received from S. Karger AG, Medical and Scientific Publishers.

In practice, these trials evaluated the effect of population-wide IPT following high-coverage active case finding. In Alaska and Greenland, tuberculosis incidence fell dramatically in both arms during the trial period (Figure 3), reflecting the impact of this active case finding and perhaps other unmeasured contemporary changes [11]. An indirect effect of the IPT intervention on transmission in control clusters could have contributed to this reduction in Alaska, where transmission between households seems likely; a similar effect seems less likely in isolated villages in Greenland.

In the Thibela TB trial (2006–2011), South African mining workforces were randomized to receive mass IPT or standard of care [13]. Here the aim was to interrupt tuberculosis transmission by mass screening linked to treatment either of tuberculosis or latent *M. tuberculosis* infection. The intervention did not reduce tuberculosis incidence or prevalence. The lack of observed effect was probably multifactorial. In some mines, the intervention did not achieve high coverage. The study had to be separated from the mine health service and management systems because of historical employee mistrust, preventing the intervention from being systematically offered to all employees. Linked to this, in some large mines intervention uptake was too slow to achieve the simultaneous coverage intended to interrupt transmission. Even in mines with near-complete coverage, the intervention effect was small and short lived. In addition, isoniazid monotherapy may have been insufficient to sterilize latent *M. tuberculosis* infection in miners (who have a high burden of *M. tuberculosis* infection and human immunodeficiency virus [HIV] infection) [14], and pretreatment loss to follow-up from the mine health service was substantial (although not atypical [15]). The epidemiological context of the Thibela TB study differed from that of the earlier trials, notably because of the very high HIV prevalence and substantial migration in Thibela; it was logistically impossible for the intervention to include other contacts (eg, households), which could be distant from the mine. Mathematical modeling based on Thibela TB data suggests that a preventive therapy intervention with high coverage, using a regimen with better sterilizing ability, and reduced pretreatment loss to follow-up, along with optimized antiretroviral therapy for HIV-positive employees, could substantially reduce tuberculosis incidence in South African gold mines [16]. However, it is sobering that, even with the considerable human and financial resources used in this rigorously conducted trial, a measurable transmission effect could not be detected.

In conclusion, when planning interventions to interrupt transmission through preventive therapy, the epidemiological and health system context must be considered. Although isoniazid has proven efficacy to reduce tuberculosis incidence at the individual level [17, 18], population-level impact may not follow unless other interventions (including active case finding linked to effective treatment) are used in combination [19], high levels of coverage are achieved, and the intervention is tailored to the local context.

## DIAGNOSTIC AND CASE FINDING INTERVENTIONS

In 2014, among all individuals with incident tuberculosis, disease an estimated 37% (>3.5 million) went unreported or, more commonly, undiagnosed [1]. This diagnostic gap varies widely by setting: for example, >80% of all estimated tuberculosis cases are detected in the Philippines, whereas in Indonesia, only one third of tuberculosis cases are notified to public health authorities.

While faster diagnosis should intuitively result in rapider treatment initiation and, hence, reduction in disease transmission, the current paradigm of passive case finding (self-referral of patients to healthcare providers) constrains the potential transmission impact of improved diagnostic tools. Patients with tuberculosis are generally symptomatic for weeks to months prior to presentation to healthcare providers [20], and they may have detectable bacilli in their sputum for many additional months before developing symptoms [21]. In the context of passive case finding and a randomized controlled trial, more-sensitive diagnostic tests (ie, the Xpert MTB/RIF assay, compared with smear microscopy and empirical treatment) shortens the time to diagnosis and effective treatment by only a few days [22].

By contrast, active case finding can detect infectious cases much earlier and, hence, potentially interrupt disease transmission. In designing active case finding interventions, it is critical to focus first in areas where more tuberculosis cases are likely to be found, including households, congregate settings (eg, prisons and mines), and healthcare facilities [23]. In both community-based active case finding studies and prevalence surveys, 20%–50% of detected cases, many of whom do not report typical tuberculosis symptoms, are smear positive[24–26]. In low-burden settings where culture for detection of *M. tuberculosis* is routinely used for diagnosis, only 10%–25% of the transmission burden is estimated to arise from smear-negative cases [27]; however, this proportion may be substantially greater in high-burden settings, where smear-negative (or intermittently smear-positive) cases may remain infectious without seeking care for very long periods. Chest radiography is a sensitive test capable of identifying these individuals [28]. Ultimately, new diagnostic tools capable of detecting tuberculosis at earlier stages will likely only have transformative impact on transmission when used in the context of broader screening and active case finding [29].

A recent randomized trial (XACT I) evaluated the feasibility and impact of the Xpert MTB/RIF assay for active case finding in South Africa and Zimbabwe, using mobile vans with on-board Xpert MTB/RIF test capacity and staffed by 3 healthcare workers each [24]. Relative to sputum smear microscopy, Xpert MTB/RIF testing was associated with a 53% increase in the number of patients initiating antituberculosis therapy. This trial demonstrates that active case finding using newer molecular tools is feasible and substantially increases the proportion of patients initiating treatment in high-burden settings. Importantly, as demonstrated in another cluster-randomized trial in South Africa (XTEND [30]), a scale-up of Xpert MTB/RIF testing is unlikely to impact mortality—or transmission—unless also accompanied by better linkage to care. Multiple ongoing studies are evaluating diagnostic tests of higher sensitivity (eg, the Xpert Ultra test [31]) and/or greater portability in the context of active case finding.

Despite these promising developments, there remain several unanswered questions regarding interventions to halt tuberculosis transmission through diagnosis and case finding. These include cost-effectiveness, feasibility and impact in different settings, optimal combination of screening tools, and characterization of tuberculosis transmission from immunological, clinical, and healthcare-seeking behavior perspectives. The relative importance of individual-level heterogeneity in infectiousness and the ability of newer tools such as cough aerosol sampling to identify individuals from whom the majority of transmission events originate also remain poorly characterized [32, 33].

With respect to drug-resistant tuberculosis, the diagnostic gap is even wider, with an estimated 70% of rifampin-resistant tuberculosis cases being undetected or unreported [1]. Given the high proportion of drug-resistant tuberculosis cases attributable to ongoing transmission [34] and the substantial delays often experienced in diagnosing and treating drug-resistant tuberculosis, the impact of improved case finding and diagnosis should be even greater. The Xpert MTB/RIF test has shortened the time to treatment initiation for multidrug-resistant tuberculosis in South Africa [35, 36] and Latvia [37], and broader implementation of a rapid molecular test, even only for passive case detection, has been projected to avert substantial morbidity and mortality due to rifampin-resistant tuberculosis in settings like India [38]. Thus, for drug-resistant tuberculosis, newer diagnostic tools could substantially reduce transmission by linking patients to appropriate therapy more quickly in settings of active or passive case finding.

In summary, early data suggest that newer molecular diagnostic tools, if combined with active case finding, may help reduce tuberculosis transmission in high-burden settings. Additional needs include trials to evaluate the effectiveness of more-sensitive and/or more-scalable diagnostic tests (especially in the context of active case finding), studies of comparative cost-effectiveness and feasibility, new tools (eg, cough aerosol samplers and novel biomarkers) to identify patients with highest transmission risk, and conclusive studies demonstrating that earlier diagnosis and treatment initiation can reduce tuberculosis burden at the population level.

## INTERVENTIONS TO REDUCE TUBERCULOSIS TRANSMISSION IN THE BUILT ENVIRONMENT

Because of infinite dilution outdoors, most tuberculosis transmission is believed to occur within buildings and other congregate settings, such as shared public transport. Exactly how to reduce tuberculosis transmission in these settings has been a challenge since the airborne nature of tuberculosis transmission was proven almost 50 years ago. That it can be done was proven in the United States and other countries during the HIV-associated resurgence of drug-resistant tuberculosis >30 years ago [39]. However, the extent of the problem, resources available, and conditions (eg, crowding) are very different in modern high-burden settings. Innovative approaches are needed.

Control strategies can be conveniently organized as source control, environmental control, and respiratory protection [40]. Source control implies understanding the sources of transmission. For decades, tuberculosis transmission control has focused on patients with known tuberculosis; thus, interventions have focused on patient separation or isolation in hospitals, cough hygiene, directional airflow, high ventilation rates, and respirator use for healthcare workers. However, the infectiousness of patients with tuberculosis falls rapidly after initiation of effective therapy, long before sputum staining for acid-fast bacilli or culture conversion [41], and patients with unsuspected tuberculosis or tuberculosis with unsuspected drug resistance ultimately represent a much greater risk of transmission [42]. This understanding has major implications for the design and use of healthcare facilities and for transmission control priorities.

Based on this knowledge, FAST (ie, find cases actively, separate cases temporarily, and treat cases effectively on the basis of rapid molecular test results), a refocused, intensified administrative approach to tuberculosis transmission control, has been proposed [43]. Ongoing FAST implementation research aims to determine the optimal screening strategy, efficient testing protocols, and appropriate process indicators, such as time from facility entry to receipt of effective treatment. While FAST implementation is achievable in many hospitals and other residential settings, application in crowded ambulatory settings is more challenging because of large numbers of symptomatic persons and the time required to make a diagnosis, even with rapid molecular testing.

In such settings, where contamination of the air is less easily prevented, use of environmental controls assumes particular importance. The World Health Organization has emphasized the role of natural ventilation—simply opening windows and doors—as highly effective and sustainable in high-burden settings [44]. Although applicable to many tropical settings, limitations include a dependence on suitable outdoor climate, air quality, and security conditions, and high rates of air change per hour may be difficult to achieve in internal corridors, which often serve as crowded waiting rooms. Alternatives to natural ventilation are few. Mechanical ventilation systems are widely used in developed countries and are effective in increasing air changes but are expensive to operate and may not achieve the same ventilation rates as natural ventilation [45]. Room air cleaners, although commonly sold, generally cannot move enough air to achieve the 6–12 equivalent air changes per hour recommended for airborne infection control. In contrast, upper-room ultraviolet germicidal air disinfection has been shown to reduce infectiousness of room air by 70% and is much more cost-effective than mechanical ventilation [46, 47]. With the advent of LED bulbs capable of generating ultraviolet light of the appropriate germicidal wavelength [48], efforts are now underway to provide guidelines, fixture specifications, and technical support to assure widespread sustainable use of this highly effective but poorly implemented technology.

Another important component of controlling tuberculosis transmission in the built environment is respiratory protection. Although they may not be worn in the presence of unsuspected cases, particulate respirators (eg, N95 masks) are nonetheless effective in helping to protect a critical population—healthcare workers who treat patients with tuberculosis. Even standard surgical masks, when worn by patients, halve the transmission of multidrug-resistant tuberculosis in the hospital setting [49] and can be given to all coughing patients. Thus, while unlikely to prevent the majority of tuberculosis transmission on its own, respiratory protection still plays an important role as part of a broader strategy of infection control in healthcare settings.

In summary, rational approaches to halting institutional tuberculosis transmission might combine FAST, natural ventilation (where appropriate), upper-room ultraviolet radiation, and respiratory protection for both healthcare staff and patients with tuberculosis symptoms. What is now required is a path to sustainable implementation. One such approach might include institutional coaching with ongoing person-to-person education, support, feedback, and follow-up. A broader evidence base of successfully implemented measures that stop tuberculosis transmission within the built environment across multiple epidemiological settings would be a major step forward.

## EVALUATING INTERVENTIONS TO HALT TUBERCULOSIS TRANSMISSION

Ultimately, it is important to demonstrate not just that interventions improve individual-level outcomes, but also whether such interventions reduce tuberculosis transmission at the population level. In estimating population-level impact, randomized trials, quasi-experimental designs, interrupted time series analyses, and mechanistic models all play a prominent role.

A fundamental design that captures population-level impacts of an intervention is the cluster-randomized trial (also known as a community-randomized trial) [50]. In designing cluster-randomized trials, there 3 important features: (1) how the cluster is defined, (2) how randomization is conducted, and (3) what outcomes to use. While a larger number of smaller-sized clusters is statistically most efficient, larger clusters more effectively capture both direct and indirect effects of interrupting transmission. In the ZAMSTAR study, for example, large clusters incorporating entire and discrete populations were chosen, resulting in 24 clusters with a combined population of 1.2 million [51]. For randomization, matched sets or stratification are often used to reduce between-cluster variation (thus maximizing power) and to balance study arms on variables that may be highly correlated with the main end point. Selecting outcomes for studies of tuberculosis transmission interruption currently involves an imperfect choice between 1 or more end points of tuberculosis prevalence, tuberculosis, *M. tuberculosis* infection incidence, and tuberculosis notifications [52, 53]. Limitations of prevalence include its dependence on disease duration and logistical difficulties in conducting prevalence surveys of sufficient size for statistical power. Prospective measurement of tuberculosis incidence is also logistically complex and may require regular sampling of cohort members. Incidence of infection is often measured using tuberculin surveys (or interferon γ release assays) in children and may not represent infection patterns among adults. Tuberculosis case notification data are often low quality and lack a suitable denominator. Ultimately, no currently used epidemiological outcome measure is perfect, and investigators must weigh the relative strengths and limitations of each.

An alternative to the parallel cluster-randomized trial is the stepped-wedge trial, where clusters are randomized as to the order of intervention implementation, such that all clusters start in the control phase and move to the intervention phase [54]. For any cluster-randomized trial, power calculations and analysis techniques must consider the clustered design, as well as any matching or stratification in the randomization [50].

When randomization is not feasible, quasi-experimental designs can estimate population-level impact by comparing outcomes (such as tuberculosis prevalence) before and after an intervention, in the same [55] or multiple [56] communities. Such studies are vulnerable to secular trends, but large and specific effects may still be persuasive. Comparison (nonintervention) communities can also be used, using a difference-in-differences analysis (or without a “baseline” measurement). In these nonrandomized studies, controlling for confounding (through matching or analysis) is critical. Process evaluation to determine plausible pathways from intervention to outcome can also support inferences [57].

One final observational approach is the interrupted time series, where repeated measurements of (often routine) data are used to assess the effect of an intervention [56]. This approach, which addresses concerns about regression to the mean, is best used where there are clearly defined periods before and after then intervention and where the outcome changes quickly after intervention implementation [58].

In some cases, empirical data cannot feasibly be collected to demonstrate the impact of interventions; reasons include intervention complexity, limited resources, time-dependent decision-making, and ethical considerations. Mechanistic (ie, mathematical) models can be useful to project intervention impact into the future, understand the mechanistic underpinnings of intervention effects, and generalize empirical findings to other settings [59]. Mechanistic models have successfully been used to describe the conditions needed to achieve tuberculosis elimination [4], understand the role of subclinical tuberculosis in determining the impact of diagnostic interventions [29], improve decision-making regarding novel tuberculosis treatments [60], and evaluate the degree to which epidemics of drug-resistant tuberculosis are driven by transmission [34]. In developing comprehensive approaches to halt tuberculosis transmission in complex epidemiological settings, it will become increasingly important to design trials and construct mechanistic models capable of evaluating not just individual interventions in isolation, but also multifaceted strategies, including prevention of progression, diagnosis and case finding, and improved infection control.

## CONCLUSION

In public health, isolated interventions rarely solve major problems; this holds true for the challenge of interrupting tuberculosis transmission. There are many potential leverage points, and effective strategies will need to direct interventions at the most important points, with the understanding that the portfolio of existing and novel tools will be wielded differentially in different epidemiological and environmental contexts. But ultimately, there is cause for optimism. There are effective interventions available to target each component of tuberculosis transmission (and the analytical tools to demonstrate their effectiveness). Furthermore, the lack of a transmission-important animal reservoir facilitates focus on human populations, behaviors, and environments. The growing enthusiasm of researchers, funders, and policy-makers to intervene against tuberculosis transmission is supported by the fresh refocus on innovative approaches mandated by the ambitious global End TB Strategy targets [1]. Although intensive and integrated approaches will be needed, we have the necessary building blocks to accomplish this goal. We must now use our existing knowledge base and analytical tools to develop new approaches to halt tuberculosis transmission globally.

## Notes

***Acknowledgments.*** We thank the workshop participants and session chairs, for valuable discussions; and Drs N. Sarita Shah and Roxana Rustomjee, for reviewing earlier versions of the manuscript; and the editorial and administrative staff of the Division of AIDS, National Institute of Allergy and Infectious Disease, particularly Ms Kathleen Muldoon, for their assistance.

***Financial support.*** This work was supported by the Division of AIDS, National Institute of Allergy and Infectious Diseases, National Institutes of Health, Department of Health and Human Services (contract HHSN272201100001G); and the Bill and Melinda Gates Foundation.

***Supplement sponsorship.*** This work is part of a supplement sponsored by the Centers for Disease Control and Prevention and the National Institutes of Health.

***Potential conflicts of interest.*** All authors: No reported conflicts of interest. All authors have submitted the ICMJE Form for Disclosure of Potential Conflicts of Interest. Conflicts that the editors consider relevant to the content of the manuscript have been disclosed.
